# Onion (*Allium cepa*) and its Main Constituents as Antidotes or Protective Agents against Natural or Chemical Toxicities: A Comprehensive Review

**DOI:** 10.22037/ijpr.2020.112773.13940

**Published:** 2021

**Authors:** Mahyar Dorrigiv, Armin Zareiyan, Hossein Hosseinzadeh

**Affiliations:** a *Department of Pharmacognosy, Faculty of Medicine, AJA University of Medical Sciences, Tehran, Iran.*; b *Public Health Department, Nursing Faculty at Aja University of Medical Sciences, Tehran, Iran. *; c *Pharmaceutical Research Center, Pharmaceutical Technology Institute, Mashhad University of Medical Sciences, Mashhad, Iran. *; d *Department of Pharmacodynamics and Toxicology, School of Pharmacy, Mashhad University of Medical Sciences, Mashhad, Iran.*

**Keywords:** Onion, Allium cepa, Protective effect, Antidote, Venom, Toxicity, Toxin

## Abstract

Onion (*Allium cepa*) is a member of the family Amaryllidaceae and one of the most widely cultivated species of the genus *Allium*. Onion has plentiful chemical compounds such as allicin, quercetin, fisetin, other sulphurous compounds: diallyl disulphide and diallyl trisulphide. Onion and its main components in specific doses have shown a lot of benefits including free-radical scavenging and antioxidant properties, anticholesterolemic, anti-heavy metals toxicity, antihyperuricemia, antimicrobial, anti-gastric ulcer, and anticancer. This study summarizes numerous *in-vitro* and animal studies on the protective effects of onion against natural and chemical toxicities. Onion and its main components can ameliorate the toxicity of chemical agents in kidney, liver, brain, blood, heart, reproductive system, embryo, pancreas through reducing lipid peroxidation, antioxidant effect, radical-scavenging, anti-inflammatory, chelating agent, cytoprotective activities, increasing protein synthesis in damaged tissues, suppressing apoptosis, as well as modulation of PKC-𝜀/p38MAPK, Wnt/beta-Catenin, ERK, JNK, p38 MAPK, Bcl-2, Bax, and NF-κB signaling pathways.

## Introduction

**Figure 1:**
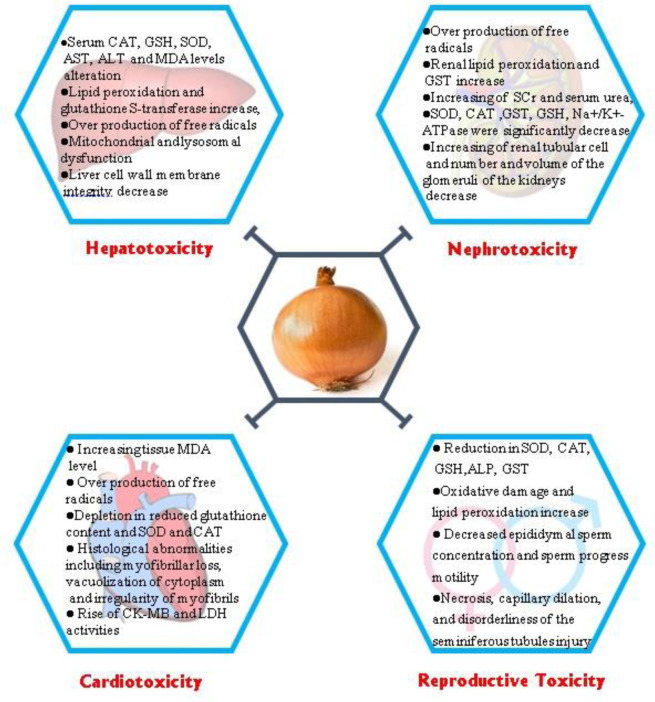
Some protective effects of *Allium cepa* against toxicity with its underlying mechanisms. CAT: Catalase; GSH: Glutathione; SOD: Superoxide dismutase; AST: Aspartate Aminotransferase; ALT: Alanine transaminase; MDA: Malondialdehyde; GST: Superoxide Dismutase; SCr: serum creatinine; LDH: Lactate dehydrogenase, ALP: Alkaline Phosphatase

**Figure 2:**
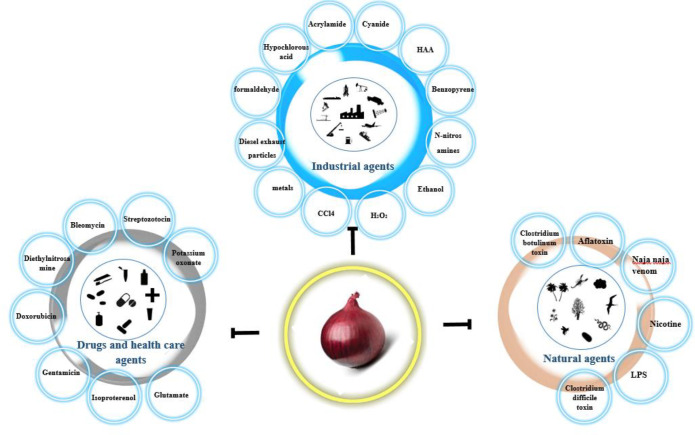
Protective effects of *Allium cepa (A. cepa) *extract against natural agents, industrial agents, and drugs and health care agents

Onion (*Allium cepa*) belonging to the family Amaryllidaceae and the genus *Allium *is a notable spice around the world, probably native from the south-west of Asia. Onion is the origin of numerous phytomolecules, such as polyphenolic substances, phenolic acids, flavonoids (fisetin, quercetin) (1-4), ascorbic acid, and sulphur compounds (5, 6) that are responsible for its color, flavor, and aroma as well as for its possible health advantages, such as its anti-toxic, anticarcinogenic properties, antiasthmatic, antithrombotic activity, antiplatelet activity, antibiotic effects, and ability to modulate the detoxification systems (7). According to various studies and research projects, onion has a lot of health benefits, such as amelioration of renal failure (8-10), antidiabetic, hypotensive, hypolipidemic properties (11-14), and lowering blood uric acid (15). Additionally, in various studies onion and its main components have a variety of protective effects in different organs and tissues including liver (1, 11 and 16), intestine (17), heart (18), testis (12, 19), kidney (9, 13), blood (20), bone marrow (21) and brain (22, 23). Other herbs and their main ingredients such as saffron, *Vitis vinifera *(24), *Curcuma longa* (25), *Cinnamomum zeylanicum* (26), *Berberis vulgaris* (27), *Nigella Sativa* (28), green tea (29), and lycopene (30) play important roles as an antidote or protecting agents against both natural and chemical toxicities. This article has reviewed the antidotal and protective effects of onion against natural toxins and chemical-induced toxicity. The effects of onion and its major components against toxicities of natural, industrial chemicals, drugs and health care products agents are introduced in Tables 1-3 and Figures 1 and 2.

## Experimental

In this review article, a comprehensive search was conducted for studies that have been published until March 27, 2019, in the following databases: PubMed, Web of Science, SciVerse Scopus, and Embase. The following medical subject headings and keywords, such as ‘*Allium cepa*’, onion, ‘protective effect’, antidote, venom, toxicity, cardiotoxin, cardiotoxic, neurotoxin, hepatotoxic, hepatotoxin, nephrotoxin, nephrotoxic, mycotoxins, aflatoxin, genotoxic, lipopolysaccharide, and toxin were used. 

**Natural agents induced toxicity**

*Aflatoxin*

Aflatoxins are secondary metabolites of certain strains of *Aspergillus flavus* and *Aspergillus parasiticus (A. parasiticus) *that have been associated with toxigenic, carcinogenic, mutagenic, and teratogenicity to different species of animals (31, 32). In a study performed on sexually mature male Sprague-Dawley rats, the effect of aflatoxin on these rats and possible antitoxic effects of onion were examined. In this study, the rats were fed contaminated aflatoxin food. The rise of ALT, AST, ALP, cholesterol, triglyceride, total bilirubin, LDH, urea, creatinine, and creatinine kinase were considered. Albumin and total protein also decreased in these rats. The co-treatment with oil-soluble extract of onion in these rats ameliorated effects of aflatoxin on serum biochemical biomarkers. Although aflatoxin almost did not affect kidney and liver GSH content, it reduced SOD activity and increased MDA levels. However, onion extract ameliorated these effects (31).

*Clostridium botulinum*

*Clostridium botulinum *(*C. botulinum*) is an anaerobic bacterium whose growth and toxin production in food causes hazardous syndrome in the human body and is responsible for several emergency admissions. Several methods have been suggested as preventive measures of its growth in the food chain. Some ingredients suggested for such use have been associated with carcinogenesis. Contamination by *C. botulinum* might be caused by different subtypes of this bacteria, such as A, B, and E which differ in response to different preventive measures. Onion oil as a preventive measure was efficient in the inhibition of *C. botulinum* type A toxin production in meat, but it fails in inhibition of type B and E toxin production (33).

*Clostridium difficile*

*Clostridium difficile *(*C. difficile*) is a healthcare-associated infection that is usually associated with antibiotic use. Two toxins are the main factors responsible for symptom contribution: toxin A and Toxin B. Aside from those two toxins, a third toxin is named binary toxin whose virulence is not fully known. Reduced efficacy and recurrence have been reported with the use of traditional treatment (metronidazole and vancomycin) against *C. difficile*. Fresh onion bulb extract has reduced the production of all these three toxigenic strains and inhibited toxin production by ≥ 40% in these strains (34).

*Lipopolysaccharides (LPS)*

LPS or endotoxins are one of the components of Gram-negative bacteria’s outer membrane, consisting of lipid and polysaccharide. While in the body, it can affect the immune system and cause septic shock and inflammation. Also, it can modulate bone remodeling by inducing bone resorption. This effect of LPS has been observed *In-vivo* and *in-vitro*. MC3T3-E1 (mouse preosteoblast cell line) cells were affected by LPS that showed promoted apoptosis. Quercetin is one of the main flavonoid components of *A. cepa* extract (2). Pre-treatment and treatment of these cells with quercetin decreased the number of apoptotic cells. With treatment, quercetin showed inhibitory effects on days 5 and 7, but the effects of pre-treatment were present after 24 h. Pre-treatment with quercetin also improved the number of calcified nodules compared to the non-treated group. The expression of osteoblast-specific genes (ALP, Runx2, OSX, and OCN) also increased in a dose-dependent manner with the pre-application of quercetin. This pre-treatment effect also extended to the caspase cascade. Pre-treatment with quercetin increased the depleted expression of B-cell lymphoma 2 (Bcl-2) and B-cell lymphoma-extra large (Bcl-XL) and decreased the enhanced expression of caspase-3, BCL2 Associated X (Bax), and cytochrome c compared to the non-treated group. With or without the presence of LPS, quercetin enhanced the expression of these mitogen-activated protein kinase (MAPK) proteins: p-ERK1/2, Wnt3, and β-catenin and done the opposite effect on p-p38 and GSK-3β protein levels. SP600125, PD98059, SB203580, XAV939, and the Wnt/β-catenin inhibitor attenuated all quercetin effects on MAPK proteins and also resulted in Bcl-2 and Bcl-XL enhancement as well as the opposite effects on caspase-3, Bax, and cytochrome c. The quercetin effects on osteoblast-specific genes were also absent after such applications. Dexamethasone application also resulted in attenuation of both LPS and quercetin effects on MAPKs and the protein expression of osteoblast-specific genes. From the data mentioned here, it can be concluded that quercetin can protect the bone against LPS induced absorption and may even improve bone formation (21). 

In BV-2 microglial cells, LPS can decrease cell viability and cause NO production. It also increases COX-2 expression at the mRNA and protein levels and proinflammatory cytokine (IL-6, TNF-α, and IL-1β) expressions. Pre-treatment with methanolic extract of *A.* *cepa* ameliorated all these effects and increased BV-2 microglial cell viability. It also promoted Bcl-2 expression which might be responsible for its cell viability improvement effects. HO-1 and NQO1 mRNA expression also increased in the presence of *A. cepa* extract, which has the potent capability to detoxify harmful compounds, combat reactive oxygen species, and directly or indirectly modulate the BBB permeability, immune system, as well as inflammatory response. *A. cepa* extract also increased catalase mRNA expression. The methanolic extract of *A. cepa* also improved cell viability in the presence of 1-methyl-4-phenylpyridinium (another toxic compound for BV-2 microglial cells) (34). 

*Nicotine*

Nicotine is a natural alkaloid with potent parasympathomimetic effects. Its main route of exposure for humans is through tobacco smoking. Nicotine was shown to exert dose-dependent degenerative changes in the lung and pulmonary tissues. Nicotine is the main risk factor for many pulmonary diseases such as lung cancer and COPD. Administration of nicotine to Sprague-Dawley adult male albino rats made their weight gain and lung protein depleted. It also increased the malondialdehyde (MDA) levels and decreased the activity of catalase (CAT), superoxide dismutase (SOD), and the levels of glutathione (GSH) and epithelial lining fluid (ELF). Administration of onion extract following nicotine was able to attenuate all the alterations made by nicotine. Though the alterations attenuated, onion extract was unable to fully reverse these effects. In histopathological studies, onion was unable to correct nicotine-induced changes including thickened interalveolar septa, congested capillaries, and massive foamy macrophages. In semithin and ultrathin sections, foamy macrophages were the main pathologic observation remained in onion treated groups (14).

*Naja naja karachiensis*

Pakistani cobra with the scientific name of *N. n. karachiensis* is one of the poisonous snakes of southern Asia. This snake is one of the Elapidae family subgroups. *N. n. karachiensis* envenomation can cause many complications such as hypotension, edema, pain, paralysis, necrosis, cardiac arrest, mucus discharge, bleeding gums, bleeding wounds, hematuria, and coagulopathy. It also can induce acute cardiac, hepatic, renal, and musculoskeletal toxicity. One of the many effects seen after this snake envenomation is hemolysis. This snake’s venom seems to destabilize the human red blood cell (HRBC) membrane. This effect may be caused by phospholipase A2 (PLA2), protease enzymes, and partly 5’-nucleotidases. The bulbs of *A. cepa* were found helpful to neutralize snake venom hemolysis*. A. cepa* was able to inhibit PLA enzyme 76% and neutralize PLA2 hydrolytic activity at a rate of 50% at all concentrations, but it failed to inhibit 5’-nucleotidases activity (35-37). Onion was able to normalize CK-MB enhanced levels after *N. n. karachiensis *envenomation (38). These effects of onion may be due to HRBC membrane stabilization (39). 

The onion was also effective against bee sting symptoms. In a study in Turkey, Halil *et al.* mentioned that one of the preferred alternative treatment methods against life-threatening events resulting from bee stings by beekeepers is applying onion on the sites of the sting (40).

**Chemical induced toxicity**

*Industrial chemicals*

*Acrylamide (ACR)*

ACR is a carcinogenic compound and is widely found in cigarette smoke, fried and baked foods, carbohydrate foods, and also cosmetics. ACR is used in various industries, such as soil coagulation, wastewater management, dye synthesis, and laboratories for gel electrophoresis (43, 44). 

The routes from which ACR causes its carcinogenic effects are not fully known. It can reduce cellular antioxidant capacity and also interfere with DNA transcription and repair. Isolated Botli fish liver cells decreased the absorbance of NR (neutral red), MTT (mitochondrial dysfunction), CV (cell wall membrane integrity), and PA (immunotoxicity) assays. Treating with onion peel extract (OPE) was able to attenuate the ACR toxic effects. This attenuating function of OPE was recorded to be a dose-dependent effect and at a dosage of 20 mg/L, it was able to wipe out these effects completely. Treatment with PEG-SOD (a superoxide scavenger) and PEG-catalase (a hydrogen peroxide scavenger) showed similar effects as OPE in acrylamide exposed liver cells which indicate hydrogen peroxide and ROS roles in acrylamide induced cell toxicity. These findings also indicate that OPE may also implicate its protective effects in liver cells by promoting antioxidant capacity or either preventing formation of ROS (16).

*Aluminum (Al)*

Al is a metal element that can cause toxic effects in different organs, mostly in the nervous system. Al can be found in manufactured foods, medicines, cheese, tea, cosmetics, and is also added to drinking water for purification purposes (45, 46). 

Aluminum with chronic uses can cause changes in behavior, motor coordination, and memory performance. Biochemical and histological changes were also observed in brain tissue. Aluminum chloride chronic exposure increases AChE activity, vacuolation in the hippocampal region (and deterioration of hippocampus), and decreases pyramidal cells, GSH, and catalase activity. Administration of *A. cepa* along with aluminum chloride, chronically, ameliorate all behavioral, histological, and biochemical changes. It also reduces abnormal aluminum deposition in brain tissue (45). Aluminum also increases SOD activity and MDA level and decrease CAT, GR, and GPx activity and levels of GSH in brain tissues. These differences are also present in gene expressions. Quercetin, an onion component, ameliorated such effects. This may indicate that these constituents possess the neuroprotective ability and can pass the blood-brain barrier and exert direct effects on brain tissue (46). Quercetin metabolites have been found to inhibit the transcription of genes such as S14, FAS, apolipoprotein CIII, and transferrin (45, 47). This inhibition might be responsible for decreased aluminum deposition in the brain, as its transfer to the brain is primarily transferrin mediated (45). 

*Arsenic*

Arsenic is a heavy metal and able to form toxic compounds. It usually contaminates groundwater and its toxicity is manifested by nephrotoxicity and renal tissue changes. Its exposure also causes Bowen’s disease, squamous cell carcinomas, melanosis, and hyperkeratosis. Arsenic exposure might increase conjugated diene effects in brain and liver tissues and decrease SOD and CAT activity as well as glutathione levels. It also results in cytochrome c protein release in the brain and liver and deposits in these tissues. While subcutaneous treatment with quercetin seems ineffective, the administration of liposomal quercetin ameliorates arsenic toxic effects and reverses histological changes via antioxidant effects. Such effects might also be due to quercetin’s ability to restrict molecular transfer in the blood-brain barrier and its chelating abilities. With such mechanisms, quercetin can decrease arsenic presence in the cellular environment (48).

*Carbon tetrachloride (CCl*_4_ *)*

In one study carried out on male adult Wistar albino rats, the protective effects of a phenolic-rich extract of red onion peels on CCl4 toxicity were examined. Carbon tetrachloride was able to increase hepatic enzymes: AST, ALT, ALP, and GGT. It also reduced albumin levels and changed lipid profiles. The TG, total cholesterol, LDL-C, and VLDL-C levels raised and HDL-c levels decreased in rats. Creatinine, uric acid, urea, and calcium levels also increased following CCl4 treatment liver and kidney MDA level increased and tissue non-protein sulfhydryl (NP-SH) and total protein synthesis capacity decreased. Pre-administration of the phenolic-rich extract of red onion peels was able to ameliorate all these effects while failed to restore pretreatment status. Its effects on all parameters were almost dose-dependent but on albumin, a dose-dependent effect was not seen (49). 

*Ethanol*

Ethanol is obtained from the fermentation of sugars in vegetables, fruits, and cereals which has played a key role in medical and industrial practices, and alcoholic beverages include beer spirits and, wines. Ethanol could affect various parts of the body including the liver, heart, brain, kidney, skeletal muscle, and pancreas (50-52). 

Fatty liver is the most common liver disease in western countries and probably the whole world. Almost 45% of individuals are affected by some degree of fatty liver, 20% of which are due to alcoholic fatty liver. Alcoholic fatty liver, in turn, can cause hepatic steatosis, fibrosis, and cirrhosis. Studies showed that alcoholic fatty liver cannot be prevented by the use of different dietary supplements except for onion and garlic oil. Feeding albino rats with 3 mL of 25% ethanol per 100 g body weight intragastrically were able to raise triacylglycerol and total cholesterol levels in serum and liver. Also, liver MDA levels reduced the following alcohol consumption. But there was no change in Liver aspartate aminotransferase (AST), alanine transaminase (ALT), and alkaline phosphatase (AlkP). Using aqueous extracts from 300 mg onions per 100 g body weight along with the named alcohol regiment decreased the rise of triacylglycerol and total cholesterol levels in serum and liver compared to alcohol-fed rats. By attenuating alcohol toxic effects in the liver, it could be suggested that onion might be able to protect against alcoholic fatty liver. Although onion extract was able to reduce these amounts, it was not able to return them to the control levels (11).

One of the components that might be involved in onion protective effects is fisetin (3, 3′, 4′, 7-tetrahydroxyflavone) at concentrations of 2–160 µg/g. Fisetin is a bioflavonoid with antioxidant, anti-inflammatory, anticancer, antihyperlipidaemic, and neuroprotective properties. Feeding male C57BL/6 mice with 5 doses of 50% ethanol orally was able to increase serum AST and ALT levels and also liver weight. Unlike liver weight, body weight decreased following ethanol use. Catalase, SOD, GSH, glutathione-S-transferase (GST), glutathione reductase (GR), vitamin C, and NQO1levels were also reduced in alcohol-affected mice. The hepatic thiobarbituric acid reactive substances (TBARS), nitrite, carbonyl content, and OH-1 expression increased. Also, the amount of MMP-9, pro-MMP-2, and active MMP-2 increased both in serum and tissue following alcohol consumption along with collagen deposition. Mitochondrial indicators such as NDH, SDH, COX, GSH, and MTT reduction assay showed a reduction in alcohol-affected mice too. Surprisingly, pretreatment with fisetin was able to attenuate all the toxic effects of ethanol-fed mice. It also was able to decrease the increased nuclear factor κB (NF-κB) levels in these mice. Fisetin also protected the liver tissue against histopathological changes seen in alcohol-treated mice (hepatocyte degeneration with vacuolization and hemorrhagic lesions, inflammatory cell infiltration, mild necrosis, and fibrosis). These findings may suggest fisetin as the onion’s responsible component in reducing the effects of alcohol on fatty liver and protecting against it (1).

*Benzopyrene (BaP)*

BaP is an aromatic hydrocarbon with potent mutagen and carcinogen activity. It is mainly a product of organic compound combustion. Its main routes of exposure are cigarette smoke, an air pollutant, and fuel combustion (53).

BaP is known for its involvement in lung and forestomach neoplasia. BaP administration can increase MNPCEs (micronucleated polychromatic erythrocytes) in mice bone marrow. Diallyl trisulfide (DAT), diallyl sulfide (DAS), allyl methyl trisulfide (AMT), and allyl methyl disulfide (AMD) all are unsaturated organosulfur compounds found in onion and garlic. These compounds contain allyl groups in their structure. DAT and AMT could prevent the formation of the forestomach adenoma in A/J mice with no effect on pulmonary adenomas. DAS showed exactly the opposite effect, with effectiveness against pulmonary adenomas, while AMD effectively inhibited tumor formation in the forestomach and lung. Interestingly, all of the compounds effective in inhibition of forestomach tumor formation were able to increase GST content. In the liver and small intestine, AMT, AMD, DAT, and DAS increased GST content too. None of these effects were observed in saturated analogs which contain propyl instead of the allyl group. DAS and AMD, both compounds with three sulfur groups, showed effectiveness against pulmonary adenoma, while disulfide compounds, DAT and AMT, have not shown such effects. This may indicate that the number of sulfur atoms may be of importance in the site they are present or effective. These findings suggest that allylic compounds present in onion and garlic may have protective effects against BaP carcinogenesis (17).

*Cadmium (Cd)*

Cd is a heavy metal that is known as an environmental contaminant usually present in low concentrations. Cd exposure has been boosted by its use in industrial sectors and also tobacco use. Cd can cause liver and kidney injury, disruption of the endocrine system, anemia, diabetes, respiratory diseases, neurological disorders, skeletal system damage, gonadotoxic and spermatotoxic effects. These toxic effects are mostly attributed to ROS formation by Cd (19, 26). 

In male albino rats, Cd exposure tends to reduce testis weight. The concentration of sperms in the epididymis, sperm motility, abnormal sperm count, and proportion of live sperm to dead sperm also reduced in the rats treated with Cd. Testis MDA level increased in Cd -exposed rats, and GSH level, SOD activity, CAT activity, and ALP activity depleted. Administering onion extract to these rats was able to attenuate sperm characteristic parameters and also improve MDA, GSH, SOD, and CAT alterations. The effects exerted on enzyme and oxidant parameters by onion extract in Cd-exposed rats showed a dose-dependent pattern. Also, GST activity changed following Cd administration. GST activity increased by both Cd and onion extract. Onion effects on the testis weight weren’t significant and it failed to reverse the cadmium effect. Onion in the absence of Cd was also able to reduce the number of abnormal sperms significantly. Such observations show that onion can protect male albino rat’s reproductive system against Cd toxicity (19). In another research performed on Sprague-Dawley rats, fresh onion extract pretreatment proved efficient in protecting against testicular weight loss. It also reversed cadmium toxic effects on sperm count, motility, morphology, testicular SOD, and CAT activity. Protection against the rise of MDA levels in the testis was also reported in these rats (54). 

Onion sulphur compounds also may have the potential in reducing Cd-induced hepatic damage. It has been observed that they can attenuate histopathological changes (focal necrosis, inflammatory cell infiltrations, and giant cell formation), serum and tissue biochemical alterations, enzymatic antioxidant capacity deterioration, lipid and protein peroxidation, and also Cd concentration. In the kidney, DTS (diallyl trisulfide) showed similar effects and exhibited cytoprotective functions. The same effects have been observed by DTS in brain tissue. DTS was also able to restore AChE and membrane-bound enzymes. Onion also was able to restore albumin and lipid profile. These effects of onion seem to be related to its effects on antioxidant capacity and lipid peroxidation (13). In the heart tissue, Cd can also cause lipid peroxidation, resulting in elevated MDA levels, which in turn could be attenuated by *A. cepa* administration. SOD, CAT, and glutathione peroxidase (GSH-Px) activities were also reduced by Cd exposure. The prevalence of apoptotic cardiomyocytes elevated too. These changes induced histopathological alterations, such as myofibrillar loss, vacuolization of cytoplasm, and irregularity of myofibrils. *A. cepa* was also able to attenuate enzymatic antioxidant alterations in the heart and reduce apoptosis. It also reduced histopathological changes caused by Cd. These changes could be due to ROS formation and lipid peroxidation which can be reversed by *A. cepa* exposure. Although *A. cepa* almost attenuated all the observed altered parameters, it seems it couldn’t prevent Cd-induced cardiac impairment (18). Following Cd administration to the rats, MDA levels increase and GSH, SOD, CAT, and GST activities deplete. These alterations compensated dose-dependently by onion extract administration. Also, onion alone tends to increase SOD, CAT, GSH, and GST levels while administered alone. This effect was present only in high doses for SOD and CAT and such effects have not been observed in accordance with AST and ALT. But the onion was able to reduce plasma AST and ALT increased levels caused by Cd. It also increased the AST and ALT activity levels in the liver tissue which again decreased by cadmium exposure. Although onion has mild but not significant effects on hepatic ALP activity, it has a significant effect on alkaline phosphatase (ALP) in the Cd -exposed rats. From what is mentioned above it can be concluded that Cd toxic effect can be almost compensated by onion extract administration (55). Cd is also a potential risk factor for prostate malignancy. It has been shown that Cd administration can decrease the weight of the prostate gland while increasing MDA levels and depleting GSH levels, SOD, and CAD activities. Although treatment with onion extract fails to retrieve prostate weight, it was successful in attenuating MDA levels and also increasing the activity of SOD and CAT along with GSH levels, dose-dependently. Onion alone also can exert the same effects on GSH levels, SOD, and CAT activity. Interestingly, both Cd and onion alone, increased activity of GST but in the presence of cadmium, onion tends to decrease GST activity and attenuate Cd effects. Cd tends to decrease total and prostatic acid phosphatase activities (ACPtotal and ACPprostatic) in the prostate and increase these amounts in plasma. Onion alone does not affect plasma ACPtotal and ACPprostatic, but it exerts Cd opposite effects in plasma. Also, coadministration of these two compounds shows that onion extract can attenuate Cd effects on ACPtotal and ACPprostatic both in plasma and prostate gland. From these observations, it can be concluded that onion can protect the prostate gland against Cd toxic effects (56). In another study, adult Wistar rats treated with Cd, showed lesser weight gain compared to control. Kidney weight also decreased in rats exposed to Cd, which tend to reverse while Cd administration concurred with onion extract administration. The kidney MDA level tends to increase by Cd administration, while onion administration alone decreased these levels. Also, Cd and onion extract co-administration showed lesser MDA levels in comparison to the Cd group alone. GSH level along with SOD and CAT activity showed a similar pattern of alteration. GSH, SOD, and CAT levels were enhanced with onion extract exposure alone in a dose-dependently pattern and were depleted after Cd administration. Also, co-treatment with both compounds showed the attenuation of Cd effects. GST levels increased after both Cd and onion administration, but the changes following onion exposure were not significant. Coadministration of both compounds again tends to show amounts similar to the control group, which itself shows the reversal of Cd effects. In accordance with renal Na^+^/K^+^-ATPase activity again onion extract and Cd showed the opposite effect. Onion enhanced renal Na^+^/K^+^-ATPase activity and Cd decreased activity. In this case, also pre-administration of onion extract in the Cd challenged group showed a tendency to the enhancement of renal Na^+^/K^+^-ATPase activity. It seems that onion extract pretreatment tends to partially compensate for lipid peroxidation and antioxidant activity depletion caused by Cd and therefore shows protective effects against Cd toxicity (8). Cadmium in male Wistar rats can cause hepatic and renal damage. Research on the effect of onion extract on cadmium toxic effects showed that *A. cepa* extract ameliorated cadmium effects on body, liver, and renal weight and also abrogate its effect on serum lipid profile (cholesterol, TG, HDL, and LDL). Although *A. cepa* was able to reduce many toxic effects of cadmium in male Wistar rats, it could not reduce the Cd’s effects on plasma testosterone (57). Also, a study concerning onion skin extract (contain phenolic compounds such as quercetin) effect on cadmium toxicity in *Saccharomyces cerevisiae* has shown similar protective effects. In this study, onion skin extract has been able to reduce ROS, OH- and O2- levels dose-dependently. It also increased GSH levels and reduced MDA levels dose-dependently. Although its effects on SOD, CAT, and GPx were dose-dependent, it was almost restricted to basal activity levels in the control group (58).

*Cyanide (CN)*

CN is a fatal poison found in several forms in the environment. It could be also found in several plants. When entering the body it can inhibit cellular oxygen utilization, induce reactive oxygen species (ROS) formation, and cause death (9). 

Several organs can be affected by CN, such as the brain, blood, liver, and kidney. A group of researchers administered CN to male albino rats to investigate the onion’s capability in protecting against sub-acute CN toxicity in the kidney. After CN exposure, relative kidney weight increased in rats. Also, it raised serum urea and creatinine level while reducing these amounts in urine. Kidney MDA levels increased while SOD, CAT, GST, and GSH activities depleted. Co-administration of onion extract with CN attenuated all these effects. In the presence of onion extract along with cyanide SOD, CAT, GST, and GSH activities improved as well as MDA levels depleted. Also, creatinine and urea levels both in blood and urine showed amounts closer to normal. CN administration also made histopathological changes, such as acute tubular necrosis. These changes were absent in high doses of onion extract (600 mg/kg) and only mild focal tubular necrosis was present in low doses (300 mg/kg) used in the experiment. From what has been observed in this study it can be suggested that onion extract as an antioxidant capacity improving agent can reduce many CN toxic effects and therefore, offer some protection against CN toxicity (9).

*Diesel exhaust particles*

It is no question today that diesel exhaust particles exert many toxic effects on the human body. They are responsible for many respiratory problems. They also might exert a toxic effect on other organ systems. There is data indicating diesel exhaust particles might also exert toxic effects on the male reproductive system. Onion powder and also its quercetin content might be of protective value alleviating such toxic effects. It has been observed that administration of onion powder or quercetin might protect daily sperm production, sperm morphological changes, and also the number of Sertoli cells (4).

*Hypochlorous acid (HCLO)*

HCLO physiologically is produced in the body via neutrophil and monocytes after inflammatory tissue injury. Upon exposure, HCLO can cause osmotic fragility and produce transient membrane pores in RBCs. This HCLO exposure may result in erythrocyte hemolysis (59). 

Onion extract application 45 min before the HCLO exposure in the *in-vitro* condition could be able to reduce hemolysis. While the effect for coppery onion (COM: Coppery Onion from Montoro) was lower than red onion (RTO: Red Tropea onion) the difference wasn’t significant. Also, the HCLO administration reduced GSH content and increased CAT activity. The application of both onion extracts was able to attenuate the HCLO effect on both parameters. Also, Onion extract was able to reduce low-density lipoprotein (LDL) oxidized levels. It seems that onion antioxidant activity has a relation with its polyphenolic content. Although ROT has greater amounts of polyphenols, it seems that in equal concentrations of polyphenols, COM polyphenols have greater antioxidant effects (59).

*Heterocyclic aromatic amine (HAA)*

HAA, potential human carcinogens, is found in cooked meat and fish (60). High heat exposure seems to be the reason for the formation of such compounds. 2-amino-3-methylimidazo[4,5-f]quinoline (IQ) and 2-amino-1-methyl-6- phenylimidazo[4,5-b]pyridine (PhIP) are two of these HAAs, found in a human dietary regiment. To apply their genotoxic effects, HAAs need to be activated by CYP enzymes. Phase II enzymes can also reduce the genotoxic activity of their metabolites. Both these compounds can be activated by V79 Chinese hamster lung fibroblasts. In *in-vitro* exposure of these cells to HAAs, onion extract showed high inhibitory effects on IQ genotoxic effects, while it was only weakly active against genotoxic effects of BaP-7,8-diol. Surprisingly, onion extract could only weakly inhibit N-OH-IQ genotoxicity (60).

*Hydrogen peroxide (H*_2_ *O*_2_ *)*

H_2_O_2_ is a colorless liquid with slight acidic properties. It is a chemically unstable molecule, naturally produced in the body with powerful oxidizing quality. Using SOD H_2_O_2_ forms and by the use of catalase, it turns to water and oxygen. While H_2_O_2_ is used by the cells in the catabolism of different molecules, its presence in other cellular spaces might be hazardous. For example, in intracellular space, it can inhibit gap-junctional intercellular communication (GJIC). Onion and especially its peel is rich in antioxidants and hypothetically can protect the body against excessive amounts of H_2_O_2_. Applying onion peel extract on WB-F344 RLE (rat liver epithelial cell line) culture in the presence of H_2_O_2_, Kim *et al.* investigated the protective effects of onion peel extract (OPE) on H_2_O_2_ inhibition of GJIC and compared it with onion flesh extract (OFE). H_2_O_2_ at a concentration of 100 *μ*M was able to suppress GJIC by 50%. Pretreatment with OPE was able to restore GJIC at 500 and 1000 *μ*g/mL, with almost complete restoration with 1000 *μ*g/mL; however, the same doses of OFE were unable to restore GJIC. It is also consistent with the polyphenol and flavonoid amounts present in these extracts. OPE contains 44 times polyphenol and 183 times flavonoid compared to OFE. In the 1,1-Diphenyl-2-picrylhydrazyl (DPPH) assay, OPE’s half-maximal inhibitory concentration (IC_50_) was 148 *μ*g/mL and showed lesser antioxidant activity compared to vitamin C. in the same test OFE showed no significant antioxidant activity, which may be indicative of the absent extracellular antioxidant activity in OFE. Kim *et al.* also repeated DPPH assay for the major polyphenols of OPE quercetin, rutin, and isorhamnetin. Both quercetin alone and the mixture of these polyphenols showed successful results in the DPPH test. Also, quercetin effect was higher compared to rutin, and isorhamnetin, and even OPE in equal doses, which highlights the importance of quercetin as the main polyphenol that exists in onion peel. H_2_O_2_ exposure also increases the density of P3 band of Cx43, which suggests the enhancement of Cx43 phosphorylation by H_2_O_2_. While OFE does not affect P3 band density, OPE dose-dependently reduced the P3 amounts, which indicating the suppression of Cx43 and ERK1/2 phosphorylation (61). In another study, the genotoxicity and lifespan effects of onion were checked in *Drosophila melanogaster,* and its DNA-clastogenic, pro-apoptotic, and cytotoxic activities were analyzed using HL60 tumoral cells. The lyophilized onion was non-genotoxic and antigenotoxic against H_2_O_2_-induced DNA damage with a positive clear dose-response effect and showed no positive effects on flies’ lifespan and healthspan (62). 

*Lead (Pb)*

Pb is a hazardous metal element with toxic neurological, behavioral, immunological, renal, ‎hepatic, gastrointestinal, reproductive, circulatory, and especially hematological effects. Pb ‎exposure during the pregnancy period is very harmful and causes a broad range of biochemical, ‎physiological, and behavioral dysfunctions in the offspring. This heavy metal is considered as one of the most common ubiquitous and industrial pollutants that are toxic even at low concentrations and exert extensive damages to the tissues. There is data suggesting that Pb ‎might exert some of its effects by causing oxidant stress indirectly. Through oxidative stress lead might decrease blood Hb, RBC, and WBC counts and ʠ-ALAD in rats. 

There are observations suggesting onion oil may alleviate such effects, even in comparison to antioxidants such as vitamin E. These superior effects extend to the protection of blood and tissue GSH level along with antioxidant enzymes, such as CAT, SOD, GR, and glutathione peroxidase against harmful lead effects. Lead is also able to increase lipid parameters, such as serum total cholesterol, LDL, cholesterol, and TAG as well as tissues. It also increases TBARS levels in tissues. In this regard, onion is as effective as vitamin E. It also might help tissues, such as the liver and kidney to regenerate (63).

*N-nitrosamines*

N-nitrosamines are an important class of chemical carcinogens that occur in human nutrition and other surroundings media and can be synthesized endogenously in the body (64). 

Administration of four N-nitrosamines, N-nitrosodimethylamine (NDMA), N-Nitrosopyrrolidine (NPYR), N-nitrosodibuthylamine (NDBA), and N-nitrosopiperidine (NPIP) to Vero cells along with onion aqueous and ethanolic extracts, showed that both onion extracts were able to reduce the cytotoxic effects of the N-nitrosamines. The aqueous extract showed only effects on NDMA and NPYR in the aqueous extract. At doses of 0.6-3 mg/mL, there was an increase in cytotoxicity but at doses of >3 mg/mL, anti-cytotoxic effects were observed. At 4-6 mg/mL, it also stimulated cell proliferation. The ethanolic extract showed effects on all four nitrosamines with dose differences as follows: >2.5 mg/mL for NPIP and >4 mg/mL for NPYR. At doses of 30-40 mg/mL, it stimulated cell proliferation. It seems that onion extracts in both forms are protective against N-nitrosamines toxicity and carcinogenicity (65).

*Formaldehyde (CH*_2_ *O)*

CH_2_O is a highly reactive natural gas compound that is flammable and soluble in water. It is the simplest form of aldehydes and is used as a disinfectant, also as an additive in cosmetic products and some other industrial purposes. CH_2_O can adversely affect cardiovascular, neurological, gastrointestinal, respiratory, and renal systems. There is evidence supporting the CH_2_O role in renal carcinogenesis. When administered to laboratory rat models, CH_2_O caused kidney volume, glomerular volume, the number of glomeruli to reduce, and blood creatinine and urea to increase. According to kidney and glomerular volume and the number of glomeruli, an alcoholic onion extract showed a reduction in CH_2_O adverse effects by increasing these parameters. But the effects of onion extract did not show a specific dose-dependent pattern or even a maximum efficient dose. The onion extract in all doses also decreased blood urea levels with the most reduction at a dose of 5 mg/kg. But in the case of creatinine, onion could only inhibit the increased parameter in 10 mg/kg dosage, and all other administered dosages (5 mg/kg, 20 mg/kg, and 40 mg/kg) it increased blood creatinine even more. Pathologically, CH_2_O caused the glomerular collapse and sclerosis via tubular necrosis and vacuolization with no interstitial tissue changes. Adding onion extract to the treatment caused the tubular and glomerular changes to fade in a dose-dependent manner but vascular changes were present in low doses, which also fade via an increase of the dose. Concerning these findings, we cannot claim a definitive protective or synergistic effect for onion alcoholic extract regarding the kidney protection against CH_2_O. Also, the effect of onion extract on the antioxidant activity of renal cells and the effect of pretreatment with onion extract remain unknown and require a broader investigation to show the effect of onion in CH_2_O toxicity (10).

**Drugs and health care products**

**(consumer products)**

*Diethylnitrosamine (DEN)*

DEN is a carcinogenic and mutagenic chemical compound soluble in water, lipids, and other organic compounds. It is used as a gasoline and lubricant additive, antioxidant, and stabilizer for industry materials. When heated it can release nitrogen oxides and also, it may damage DNA integrity by alkylation. Also, DEN is a known liver carcinogen. To investigate onion organosulfur compounds’ potency in protecting the liver against DEN a group of researchers designed a study. Bodyweight in DEN-treated rats decreased; however, dihydropyrimidine dehydrogenase (DPD), diallyl trisulfide (DAT), and PS prevented this effect. But relative liver weights depletion increased with diallyl disulfide (DDS), dipropyl sulfide (DPS), diallyl trisulfide (DAT), allyl methyl trisulfide (AMT), methyl propyl disulfide (MPD), propylene sulfide (PS), and diethyl disulfide (DMD) and decreased with DPD. Glutathione S-transferase placental form (GST-P)- positive areas increased with DAS, AMS, and DPS treatment with the most efficacy seen in DPS treatment (66). The same group investigated different organosulfur (DPT, DMT, AM, and IAIE). Bodyweight was not significantly decreased in the DEN-treated rats, exposed to DPT and DMT, and also liver weight increased with all organosulfur but the change with DMT, AM, and IAIE was only significant compared to DEN control group. Numbers and areas of GST-P-positive foci per unit area of liver sections after DEN initiation, GST-P positive areas and numbers for IAIE increased significantly. In the case of DPT and AM only numbers of GST-P-positive foci improvement were significant, which suggest that these compounds may increase the probability of liver tumor formation (67).

*Doxorubicin (DOX)*

DOX is an antibiotic agent widely used in oncologic treatments. It is of use against blood neoplasms, such as leukemia, lymphoma, and some solid tumors. DOX exerts its effects via blocking the cell cycle and inducing apoptosis. DOX also has some adverse effects such as cardiotoxicity and hepatotoxicity. These adverse effects seem to be the result of ROS formation and imbalance in cellular antioxidant activity due to DOX exposure. Using Sprague–Dawley rats as animal models, a group of researchers studied DOX hepatotoxicity and *A. cepa* protective effects against it. Following DOX administration, ALT and AST levels rose but with *A. cepa* pretreatment, ALT and AST levels declined even below the control group stats. Hepatic tissue MDA levels also rose and the levels of GSH and activities of SOD and GSH-Px reduced. In all these parameters, although *A. cepa *extract was unable to reverse the changes, it successfully attenuated the alteration. It also protected against the histopathological changes seen with DOX administration: congestion and thrombosis of central vein, degeneration and pleomorphism in hepatocytes, cytoplasmic eosinophilia, parenchymal necrosis, the proliferation of biliary duct, focal necrosis, and inflammation in portal space. Finally, it could be said that even though *A. cepa* can’t make a setback for DOX hazardous effects, it can attenuate these effects and offers some amounts of protection against DOX hepatotoxicity (68).

*Gentamicin*

Gentamicin is a famous aminoglycoside antibacterial agent with potent and widespread uses. The most restricting side effect of this drug is nephrotoxicity. The nephrotoxicity caused by gentamicin was manifested by tubulopathy. In a study, the protective effects of *A. cepa* on gentamicin-induced nephrotoxicity in Sprague Dawley rats were investigated. Gentamicin administration caused the bodyweight to deplete and kidney weight to increase. The administration of ethanolic extract of *A. cepa* (EEAC) dose-dependently attenuated these effects of gentamicin. Also, EEAC reduced serum creatinine which increased by gentamicin dose-dependently. The serum total protein levels increased with gentamicin exposure. EEAC once again attenuate this effect. Histopathologically, the group affected by gentamicin showed epithelial lining degeneration, faded by EEAC administration. From these results, it can be concluded that onion ethanolic extract can prevent gentamicin-induced nephrotoxicity (69).

*Glutamate (Glu)*

Glu is a neurotransmitter by which nerve cells send their signals to other cells. Studies suggest that glutamate can induce oxidative stress that can activate mitogen-activated protein kinase (MAPK) pathways. These pathways interact with many cellular processes, such as cell growth, differentiation, inflammation, and cell death. Glu at a concentration of 5 mM can decrease cell viability significantly. Also, cell apoptosis and ROS formation in rat hippocampal cell cultures increased significantly after exposure to 10 mM of Glu. As it has been confirmed Glu could induce a rapid Ca^2+^ influx. As a result, Glu increased the levels of calpain expression and reduced spectrin. As decreased JC-1 was observed in the cell cultures it can be concluded that Glu also disrupts mitochondrial membrane integrity. Also, the Bcl-2 and Bid levels decreased in the studied HT22 cells and Bax levels showed a slight increase. Pretreatment with quercetin increased HT22 cell viability and cell apoptosis dose-dependently. It also reduced the calpain level and increased spectrin amounts, and inconsistency with these observations, it prevented JC-1 depletion and attenuated changes in Bcl-2, Bid, and Bax. Cytochrome C release also decreased with quercetin pretreatment. In concern of MAPKs phosphorylation, the observations showed phosphorylated ERK, JNK, and p38 after Glu exposure, which is also prevented by quercetin pretreatment directly or indirectly. The data mentioned above are indicative of quercetin high potential in preventing neural cell apoptosis and ROS damage in these cells, and thus inducing protecting effects in the presence of Glu (22).

*Isoproterenol (ISO)*

ISO is a synthetic non-selective β adrenergic agonist drug. ISO due to disturbing physiological balance between formation of free radicals and antioxidative defense system causes myocardial infarction in rats (70).

ISO induces ROS formation and lipid peroxidation. It can affect the myocardium and cause stress oxidative. Male albino rats affected by ISO showed decreased systolic arterial pressures (SAP), diastolic arterial pressures (DAP), and mean arterial pressures (MAP) and also heart rate (HR) as well as rate change pressure of +/- left ventricular delta pressure/delta time (LVdP/dt). It also elevated left ventricular end-diastolic pressure (LVEDP). Also TBARS level enhanced while creatine kinase (CK-MB) and lactate dehydrogenase (LDH) amounts in myocardium diminished. After ISO exposure, the SOD and CAT activity depleted and GSH showed no alteration. To prevent such adverse effects cycloaliin predministered to the animal models. Cycloaliin (CYC) is one of the onion’s cyclic sulfur-containing compounds. CYC was able to elevate SAP, DAP, MAP, + and - LVdP/dt, and also LVEDP levels. These changes were almost dose-dependent. It also attenuated the CK-MB, LDH, and TBARS alterations, (improved CK-MB and LDH levels and TBARS levels decreased). CYC improved CAT and SOD activity. Also, CYC elevated GSH levels. Histopathologically, the affected rat’s myocardium showed degraded cardiac fibers, with extensive subendocardial necrosis, and intracellular leakage with prominent myocellular edema. The low doses of CYC were unable to induce any prominent changes in this histopathological view, but in high doses, it protects myocardial fibers and there was no sign of inflammation, necrosis, or edema (71).

*L-Buthionine sulfoximine (BSO)*

BSO is an irreversible γ-glutamylcysteine synthase (the rate-limiting enzyme for GSH synthesis) inhibitor. By reducing the GSH amount in the brain, BSO can induce oxidative stress. It can also induce cell death via the activation of PKC-𝜀. After treating the rat’s 14-day embryo’s cortical cells culture, the LDH, ROS levels along with the number of TUNEL positive cells increased. Pretreatment with *A. cepa* extract (ACE) and its derivative polyphenol quercetin (QCT) caused a reduction in all these alterations in a dose-dependent manner. The effects seen by QCT were slightly greater. Also, ERK1/2 phosphorylation increased and p38MAPKs phosphorylation decreased. While ACE, QCT, and SB202190 were all able to reduce p38MAPKs phosphorylation, U0126 was not successful in increasing ERK1/2 phosphorylation, whereas ACE and QCT increased these amounts. Unlike U0126 treatment, with the administration of SB202190 LDH, ROS alterations and the number of TUNEL positive cells attenuated, while both these matters were unable to recover ERK1/2 status. Co-administration of U0126 with ACE or QCT attenuated the antioxidant effects of ACE and QCT, which is indicating ERK1/2 phosphorylation has effect on ACE and QCT antioxidant activities. Also, QCT and ACE pretreatment decreased the amounts of PKC-𝜀 translocation (an increase in membrane fractions and a decrease in cytosolic fractions), induced by BSO. Also, the administration of 𝜀V1-2 showed the same effects. It also inhibited p38MAPK phosphorylation, LDH release, ROS accumulation, and reduced the number of TUNEL-positive cells. These data suggest the involvement of PKC-𝜀/p38MAPK signaling pathway in antioxidant activity of both ACE and QCT (2, 72 and 73). 

*Potassium oxonate*

Potassium oxonate is a uricase inhibitor that can induce the hyperuricemic state seen in gout disease. To treat such conditions various drugs are used. Allopurinol, febuxostat (XO inhibitors), probenecid, sulfinpyrazone, and benzbromarone (uricosuric drugs) are some of the drugs used to treat these conditions. While potassium oxonate was applied intraperitoneally on male Sprague-Dawley rats, it increased uric acid significantly. The application of both allopurinol and onion juice was able to reduce the blood uric acid levels. This effect seen with onion juice was in a dose-dependently manner. The final uric acid levels after the application of allopurinol and onion juice were almost similar. Also, the total antioxidant status in the rats receiving onion juice and allopurinol was increased. The enhancement seen with onion was higher than allopurinol. The restoration of total antioxidant status might be partly due to the restoration of the uric acid antioxidant effect itself when reversing to normal levels. But the higher antioxidant effect of onion might be due to its antioxidant qualities. Due to XO activity and the formation of ROS during the conversion of hypoxanthine to xanthine, the hyperuricemic condition might result in liver and kidney damage. The liver damages caused by this hyperuricemic state attenuated with onion juice treatment, but the effects of allopurinol to protect liver tissue seem to be higher than those of onion juice. Moreover, in hyperuricemic conditions renal arterial resistance may increase and renal blood flow diminished resulting in renal vascular damage. Onion’s juice failed to exert any significant change in renal histological changes (15).

*Streptozotocin (STZ)*

STZ is an antibiotic and antineoplastic agent that is produced by *Streptomyces achromogenes*. It is especially toxic to pancreatic beta cells and used to treat metastatic pancreatic islet cell carcinoma (12, 75).

Its mechanism of action is not fully known, it probably inhibits DNA synthesis and interferes in NAD, NADH reactions, and gluconeogenesis. STZ can be used to induce diabetes mellitus in Adult male Wistar rats. STZ decreased the body weight of rats. Also, the level of testosterone hormone decreased and blood glucose levels increased. With the administration of onion seed extract in doses of 200 and 400 mg/kg, the testosterone levels decreased further but the bodyweight gain was recovered. Furthermore, the blood glucose enhancement is almost restored to normal value. Histologically, STZ diabetic rats showed degenerative changes such as degenerated and atrophied seminiferous tubules with no spermatogenic series and sperms in the tubular lumen and vacuolization and exfoliation of germ cells into the lumen. With 200 mg/kg onion seed extract administration the changes were milder but in 400 mg/kg no improvement was observable. In addition, STZ induced diabetic rats showed a decreased number of germ cells especially spermatid and spermatozoa cells. With 200 mg/kg treatment the number of these cells increased significantly compared to the only STZ treated rats. 400 mg/kg onion seed administration again failed to increase the number of germ cells significantly except spermatid cells. Although volume density changes of the lumen were insignificant in STZ treated rats, the onion seed administration attenuated these changes. Two hundred milligrams per kilogram of onion seed extract also increased seminiferous tubular diameter (STD), seminiferous luminal diameter (SLD), and epithelium thickness (ET). For 400 mg/kg dosage only the changes in SLD were significant (12).

*Bleomycin (Ble)*

Ble is a radiomimetic agent that is S-phase-independent and can induce chromosomal damage in all stages of cell proliferation which is employed commonly in the treatment of testicular germ cells and tumors Hodgkin lymphoma (76). 

Ble can conduct its effects via ROS formation and DNA damages that can lead to oxidative stress, mitochondrial leakage, and apoptosis. Ble can reduce peripheral blood mononuclear cell viability. Micronucleated cytokinesis-blocked (MNCB) cells and the frequency of MNCB increased dose-dependently after Ble exposure. In comet assay, it has been shown that Ble can cause DNA damage and increase tail moment, dose-dependently. Onion extract alone decreased cell viability itself; however, it increased cell viability when it was treated before Ble administration. It also decreased micronuclei (MN) frequency in cytokinesis-block micronucleus assay (CBMN) assay and DNA damage in the comet assay. These effects observed in onion extract could be attributed to its antioxidant scavenging behavior. Regardless of pathways that may be involved, these findings indicate that onion is able in protecting against Ble genotoxicity and potentially radiation induce genotoxicity (20).

*Onion toxicity*

*Allium *genus includes over 500 species. Garlic and onion are two important species with widespread use in this genus. Onion is formed of polyphenol, flavonoids, fructooligosaccharides (FOS), thiosulphinates, and other sulfur compounds that contribute to its rich antioxidant effects. It is believed that onion may have preventive potencies against tumor promotion, CVD, and aging. Feeding male Fischer 344 rats with three different onion regiments: onion by-product, onion extract (rich in FOS), and onion residue resulted in different outcomes. 85% of the sugars, 88% of the fructans, and 91% of the flavonoids of the onion by-product were found in onion extract and 9-15% that remains can be found in onion residue, which is consist of the remaining parts after extraction. All three onion regiments resulted in Hb depletion (the effect with extract was higher). Furthermore, onion extract increased GR and GPx1 activities in erythrocytes and decreased GPx1 activity in the liver. There was almost no gene expression difference detected (hepatic Gr, Gpx1, Cat, and Alas1). Only onion residue upregulated gene expression of hepatic Gclc significantly and Nqo1 not significantly. Also, no DNA damage was detected in the fed rats. All three onion regimens resulted in a caecal pH decrease but no weight or transit time changed., An increase of β-glucosidase (BGL) and β-glucuronidase (GUS) activities were detected in the caecum. The enhancement of propionate and butyrate formation in the caecum with all three onion groups was observed. This effect was stronger with the onion residue regiment (77). 

In another study, 55 Wistar rats were used to examine the effects of *A. cepa* on spermatozoa morphology and characteristics. The methanol extract of red cultivar *A. cepa* was administered to these rats in different dosages also compared with the control group: 100 mg/kg, 200 mg/kg, 400 mg/kg, 800 mg/kg, and 1200 mg/kg. Spermatozoa concentration in the rats exposed to onion extract was reduced dose-dependently, while the reduction in motility varied in different doses. Furthermore, administration of *A. cepa* increased the number of Tailless-head morphological abnormalities between Wistar rats, with its peak in 800 mg/kg (78). Hemolysis is another effect associated with onion toxicity. This effect has been observed in different farm animals. Onion in toxic amounts might cause methaemoglobinaemia and hemolytic anemia with Heinz body formation in these animals. This illustrates decreased Packed cell volume, anorexia, hemoglobinuria as well as histological changes, such as marked periacinar necrosis, vacuolation of hepatocytes in mid-zonal areas, and a hemoglobinuric nephrosis with minimal interstitial mononuclear cell reaction have been reported (79). In another study on dogs, they have illustrated similar signs such as hematuria, anorexia, and inactivity. In these dogs rise of mean corpuscular volume, mean corpuscular hemoglobin, and WBC also a fall in hemoglobin were reported. An increase in reticulocyte count and Heinz body count and direct bilirubin level was also observed. G6PD activity reduced in these dogs progressively till day 5, which was accompanied by the highest concentration of H_2_O_2_ and MDA. An increase in GSH and CAT (responsible for H2O2 degradation) activity on day 5 was also observed. the erythrocyte deformity index was at its highest on day 5 which was correlated to MDA levels. It has been hypothesized that n-propyl disulphide, sodium n-propylthiosulfate, S-methyl, and S-propenylcysteine sulphoxides (SMCO and SPCO) are the compounds responsible for such effects (80). These compounds can be broken down into various sulphides in the animal body. It also has been observed that onion extract possesses more cytotoxic effects than any of its compounds alone, which may implicate that Onion extract’s cytotoxic effect might be the result of the synergistic action of several compounds (81). Sodium n-propylthiosulfate can cause a reduction in G6PD activity which may lead to GSH fall and lead to increase H_2_O_2_ and finally the formation of MDA through peroxidation. These events might lead to the oxidization of sulfhydryl groups of HGB and the formation of Heinz body. At last, this chain of oxidative stress might result in erythrocyte deformity, membrane fluidity, and shortened erythrocyte life span. Although it is considered that onion is a potent antioxidant, it seems that onion exerts its toxic effects by oxidative stress. Although onion extract might be of toxic qualities, onion coat and natural colorant extracted from onion have been reported to be safe in regular dosage (82). In another study treatment with methanolic extract of *A. cepa *showed no effect on the viability of N 27-A microglial cells (41).

**Table 1:** Protective effects of onion and its main constituents against natural agents induced toxicity

**Agent**	**Type of toxicity**	**Type of study**	**Study design and dose of garlic/ garlic component evaluated**	**Effect demonstrated**	**References**
Aflatoxin	Heart, liver, and kidney toxicities	*In-vivo*, male Sprague-Dawley rats	Ethanolic onion extract (2 weeks, 5 mg/kg, p.o.)	The protective role of these extracts may be due to their rich content of organosulfur compounds, which act as a precursor of GSH, which conjugates with aflatoxin-epoxide and results in the inhibition of epoxide binding to DNA	(31)
*Clostridium botulinium*	-	*In-vitro, *Meat slurry	onion oil (45 mg) was dissolved in miglyol to a final volume of 1.0 mL	Onion oil reduces Toxin concentration produced in meat slurry systems by *C. botulinum *type A, B, and C	(33)
*Clostridium difficile*	Cell toxicity	*In-vitro*, the Vero (African green monkey kidney) and HT-29 (human colon carcinoma)	Fresh onion bulb extract	Fresh onion bulb extracts reduced toxin production and activity significantly.	(34)
Nicotine	Lung toxicity	*In-vivo*, Sprague-Dawley adult male albino rats	Onion extract (18 weeks, p.o.)	Antioxidative and anti-lipid peroxidative mechanisms.	(14)
LPS	Bone marrow toxicity	*In-vitro*, MC3T3-E1 cells,	Quercetin	Restored LPS-suppressed bone mineralization and the mRNA and protein expression levels of osteoblast-specific genes such as Osterix (OSX), runt-related transcription factor 2 (Runx2), alkaline phosphatase (ALP) and osteocalcin (OCN)	(21)
LPS	Cell toxicity	*In-vitro*, BV-2 microglial cells, N27-A cells	Methanol extract of onion	Onion extract takes protective action against LPS and MPP+, and upregulates the antioxidant enzymes that could potentially be used in the therapy of neurodegenerative diseases	(41)
*N. n. karachiensis* toxin	Hematotoxicity	*In-vitro* , hen’s egg yolk mixture	Ethanolic onion extract (0.1 to 0.6 mg/mL)	Neutralize snake venom proteins due to the abundance of miscellaneous secondary metabolites.	(35)
*N. n. karachiensis toxin*	Heart, liver, kidney, and musculoskeletal toxicities	*In-vivo*, male rabbits	Ethanolic onion extract (100 mg/kg, sc)	Secondary metabolites pose a hindrance in the binding of different snake venom enzymes to their potential targets.	(36)
*N. n. karachiensis* toxin	Hematotoxicity	*In-vitro*	Methanolic onion extract	Secondary metabolites created disturbances in binding of 5í-nucleotidases to their receptor(s), therefore resulted in the recovery of different toxicities.	(39)
*N. n. karachiensis* toxin	Hematotoxicity	*In-vitro*, hen’s egg yolk mixture	Ethanolic onion extract (0.1 to 0.6 mg/mL)	Neutralize snake venom proteins due to the abundance of miscellaneous secondary metabolites.	(37)
Histamine	Gastrointestinal toxicity	*In-vivo*, rats	allylsulfide, allyldisulfide and quercetin	By inhibition of gastric secretion stimulation effect of histamine.	(42)

**Table 2:** Protective effects of onion and its main constituents against industrial chemicals induced toxicity

**Agent**	**Type of toxicity**		**Type of study**		**Study design and dose of garlic/garlic component evaluated**		**Effect demonstrated**		**References**
Acrylamide	Hepatotoxicity		*In-vitro*, Bolti fish (Tilapia nilotica) liver cells		Onion peel powder		Direct ROS scavenging activity		(16)
Aluminum	neurotoxicity		*In-vivo*, male albino rats		Quercetin (100 mg/kg, 2 months, p.o.)		significant reduction in free radical concentration and induction of the activity and gene expression of the brain antioxidant enzymes		(46)
Aluminum	neurotoxicity		*In-vitro, *Swiss albino male mice		Onion hydroethanolic extract (50, 100 and 200 mg/kg/day, 2 months, p.o.)		*A. cepa* reduced aluminium deposition in the brain, which could be the major mechanism responsible for its neuroprotective potential besides inhibiting the ROS production		(45)
Arsenic	Hepatotoxicity, neurotoxicity		*In-vitro,* Adult female Swiss albino rats		Liposomal quercetin (2.71 mg /kg BW, twice a week for 4 months, S.C.)		Liposomal quercetin reduce arsenic deposition in the brain and liver		(48)
Carbon tetrachloride	Nephrotoxicity, hepatotoxicity		*In-vitro, *rats		hydro-acetone phenolic-rich extract of red onion peels (50 and 100 mg/kg/rat, 17 days, p.o)		The extract possesses mainly various phenolic phytochemicals which can diminish the free radicals-induced oxidative stress		(49)
Diesel exhaust particles	reproductive toxicity		*In-vivo*, BALB/c male mice		freeze-dried onion powder, 0.5% w/w of the daily diet, 5 weeks, p.o)		effects depend on the aryl hydrocarbon receptors antagonistic function		(4)
Ethanol	Hepatotoxicity		*In-vivo*, Male Sprague-Dawley rats		Onion wine extract (1 mL/d, 6 weeks, p.o.)		Ameliorate ethanol-induced fatty liver by lowering hepatic and blood lipid levels		(11)
Ethanol	Hepatotoxicity		*In-vivo*, male C57BL/6 mice		Fisetin (5 mg/kg, 8 consecutive days, p.o.)		Restored the alcohol-induced histological alterations, antioxidants defenses, NF-κB expression and stabilized the matrix metalloproteinases (MMPs) activity in the liver tissue		(1)
Benzopyrene	Gastrointestinal toxicity		*In-vivo*, female A/J mice		Allyl methyl trisulfide (AMT), allyl methyl disulfide (AMD), diallyl trisulfide (DAT), and diallyl sulfide (DAS)		Induced increased glutathione S-transferase (GST) activity		(17)
Cadmium	Reproductive toxicity		*In-vivo*, Adult male albino Wistar rats		Onion extract (0.5 mL/100 g BW/day, 7 days, p.o.)		Reducing lipid peroxidation and increasing the antioxidant defense mechanism.		(19)
Cadmium	Cardiac toxicity		*In-vivo*, male Sprague-Dawley rats		Onion extract (1 mL, 30 days, p.o.).		Possibly through antioxidant and anti-apoptotic activity.		(18)
Cadmium		Hepatotoxicity		*In-vivo*, Adult male albino Wistar rats		Onion extract (0.5 mL/100 g BW/day, 7 days, p.o.)		Reduced lipid peroxidation and enhanced antioxidant defense system.	(55)
Cadmium		Reproductive toxicity		*In-vivo*, Adult male albino Wistar rats		Onion extract (0.5 mL/100 g BW/day, 7 days, p.o.)		Enhancement of antioxidant status.	(56)
Cadmium		Nephrotoxicity		*In-vivo*, Adult male albino Wistar rats		Onion extract (0.5 mL /100 g BW/day, 7 days, p.o.)		Protective effects via the reduction in LPO and enhanced antioxidant defense.	(8)
Cadmium		Hepatotoxicity, cardiovascular toxicity		*In-vivo,* male Wistar rats		Onion extract (1 mL/100 g BW, 8 weeks, p.o.)		Onion extract protected against Cd-induced atherosclerotic condition via a mechanism dependent on lipid peroxidation but not plasma testosterone level	(57)
Cadmium		Reproductive toxicity		*In-vivo, *Adult male Sprague-Dawley rats		1 Onion extract (mL/100 g BW, 8 weeks, p.o.)		Onion extract attenuated the derangement of lipid peroxidation profile in testicular tissues caused by CdSO4 exposure	(54)
Cadmium		Cell toxicity		*In-vitro,* yeast strain NCPF 3178		Onion skin extract		Onion skin extract protected yeast cells from the occurrence of oxidative stress induced by cadmium	(58)
Cyanide		Nephrotoxicity		*In-vivo*, rats		Onion (600 and 300 mg/kg BW/day)		Reduced lipid peroxidation in the kidney and increased antioxidant status	(9)
formaldehyde		Nephrotoxicity		*In-vivo*		Hydro-alcoholic extract of onion (5, 10, 20 and 40 mg/kg/day14 days, p.o.)		Reduced lipid peroxidation in the kidney and increased antioxidant status	(10)
Hypochlorous acid		Hemotoxicity		*In-vivo*, human erythrocytes		Onion extract		Protective effects against oxidative damage.	(59)
Heterocyclic aromatic amines		Genotoxicity		*In-vitro*, immortal mammalian cells		Onion juice		By the inhibition of activating enzymes (CYP enzymes)	(60)
Hydrogen peroxide		Hepatotoxicity		*In-vitro*, WB-F344 rat liver epithelial cells		Onion flesh extract		Antioxidant activities	(61)
N-nitrosamines		Nephrotoxicity		*In-vitro*, Vero cells (African green monkey kidney cells)		Onion Aqueous extracts		Antioxidant activities	(65)
Lead		Hepatotoxicity, cardiotoxicity, and nephron toxicity		*In-vivo*, Male Sprague-Dawley albino rats		Polar fraction of onion oil (100 mg/kg/BW, 1 month, p.o.)		Antioxidant activities	(63)

**Table 3:** Protective effects of onion and its main constituents against drugs and health care products (consumer products) induced toxicity

**Agent**	**Type of toxicity**	**Type of study**	**Study design and dose of garlic/ garlic component evaluated**	**Effect demonstrated**	**References**
Potassium oxonate	Nephrotoxicity hepatotoxicity	*In-vivo*, Male Sprague-Dawley rats	Onion juice (10.5 g/kg/day, 14 days,p.o.)	Antioxidative mechanisms	(15)
Streptozotocin	Reproductive toxicity	Adult male Wistar rats,	*A. cepa* (Onion) seeds (AC) extract (200 or 400 mg/kg/day, 28 days, p.o.)	Antioxidative mechanisms.	(12)
Bleomycin	Cytotoxicity	*In-vitro*, human lymphocytes,	onion extract(10 and 20 μl/ml)	Antioxidant activities	(20)
Diethylnitrosamine	Hepatotoxicity	*In-vivo*, male F344 rats	Organosulfur compounds	Increased cell proliferation with increased poly‐amine biosynthesis	(67)
Diethylnitrosamine	Hepatotoxicity	*In-vivo*, male F344 rats	Organosulfur compounds	Increased cell proliferation with increased polyamine biosynthesis	(66)
Doxorubicin	Hepatotoxicity	*In-vivo*, male Sprague Dawley rats	Onion extract (1ml, 14 days, p.o.)	Enhancement of antioxidant status	(68)
Gentamicin	Nephrotoxicity	*In-vivo*, male Sprague Dawley rats	Ethanolic extract of extract of onion (200 and 400 mg/kg, 14 days, p.o.).	Protecting the kidney from the oxidative stress	(69)
Glutamate	CNS toxicity	*In-vitro*, HT22 cells,	Quercetin	Reducing both intracellular ROS overproduction and glutamate-mediated Ca(2+) influx.	(22)
L-Buthionine sulfoximine	Neurotoxicity	*In-vitro, *corticalneuronal cells derived from mouse embryos	Onion extract	inactivation of PKC-𝜀 induced by phosphorylating ERK1/2 is responsible for the neuroprotective effect of Onion extract against BSO-induced oxidative stress	(2)
Isoproterenol	Cardiotoxicity	*In-vivo*, male albino Wistar rats	Cycloalliin) 10, 20 and 30 mg/ kg, 30 days, p.o.)	Antioxidant property reduced harmful effects of ROS generation	(71)

## Conclusion

Onion and its main components in the present review article manifested antidotal and protective effects against natural and chemical toxicities through *in-vitro* and *in-vivo* studies.

Under data from various studies, onion, and its main components have a significant protecting impact against environmental, industrial, natural, and agricultural toxins including environmental pollutants (acrylamide, carbon tetrachloride, benzopyrene, and cyanide), heavy metal (cadmium), LPS, nicotine, glutamate, and also noteworthy protecting effects against toxicity of some drugs such as acetaminophen, gentamycin, aspirin, bleomycin, doxorubicin, cyclophosphamide, streptozotocin, and ISO in various tissues. Different mechanisms such as lowering lipid peroxidation, increasing the antioxidant defense mechanism, radical scavenging, anti-inflammatory, chelating agent, cytoprotective activities, increase protein synthesis in damaged tissues, suppressing apoptosis, the modulation of PKC-𝜀/p38MAPK, Wnt/beta-catenin, ERK, JNK, p38 MAPK, Bcl-2, Bax, NF-κB signaling pathways, and cytochrome c are involved in onion protective and antidotal effects. Although due to its different functions in various tissues, it would also be necessary to study other possible mechanisms, such as AKt and Nrf2 pathways that are involved in the benefits of onion and the major components. Overall, onion and its main components can be considered as potentially therapeutic and protective agents against different toxicities. To confirm the favorable impacts of onion, it is proposed to verify different experimental records by clinical trials on humans.
